# Supplementary material for: Ultra-Processed Food Consumption as a Risk Factor for Gastrointestinal Cancer and Other Causes of Mortality in Southern Italy: A Competing Risk Approach
Source: Nutrients. 2024 Jun 23;16(13):1994. doi: 10.3390/nu16131994 (PMC11243523; doi:10.3390/nu16131994)
Supplement: Supplementary file 1 [file nutrients-16-01994-s001.zip › nutrients-3053435-supplementary.pdf]

Supplementary materials

Table S1. Distribution of Gastrointestinal Cancers by Quartiles of Daily UPF Consumption.

Gastrointestinal Cancers	Quartile of UPF consumption (g/day)				
	<80	80-140	141-240	>240	All
	N (%)	N (%)	N (%)	N (%)	N (%)
N	43)	34	13	15	105
C14	1 (2.33)	1 (2.94)	1 (7.69)		3 (2.86)
C16	3 (6.98)	4 (11.76)	2 (15.38)		9 (8.57)
C18	9 (20.93)	9 (26.47)	3 (23.08)	1 (6.67)	22 (20.95)
C19	1 (2.33)				1 (0.95)
C20	5 (11.63)	1 (2.94)	1 (7.69)	1 (6.67)	8 (7.62)
C22	18 (41.86)	11 (32.35)	2 (15.38)	3 (20.00)	34 (32.38)
C23		2 (5.88)		1 (6.67)	3 (2.86)
C25	3 (6.98)	6 (17.65)	4 (30.77)	7 (46.67)	20 (19.05)
C26	3 (6.98)			2 (13.33)	5 (4.76)

Percentages calculated for the column. UPF: Ultra Processed Food; C14: Malignant tumor of other and ill-defined sites of the lip, oral cavity, and pharynx; C16: Malignant tumor of the stomach; C18: Malignant tumor of the colon; C19: Malignant tumor of the recto sigmoid junction; C20: Malignant tumor of the rectum; C22: Malignant tumor of the liver and intrahepatic bile ducts; C23: Malignant tumor of the gallbladder; C25: Malignant tumor of the pancreas; C26: Malignant tumor of other and ill-defined organs of the digestive system.

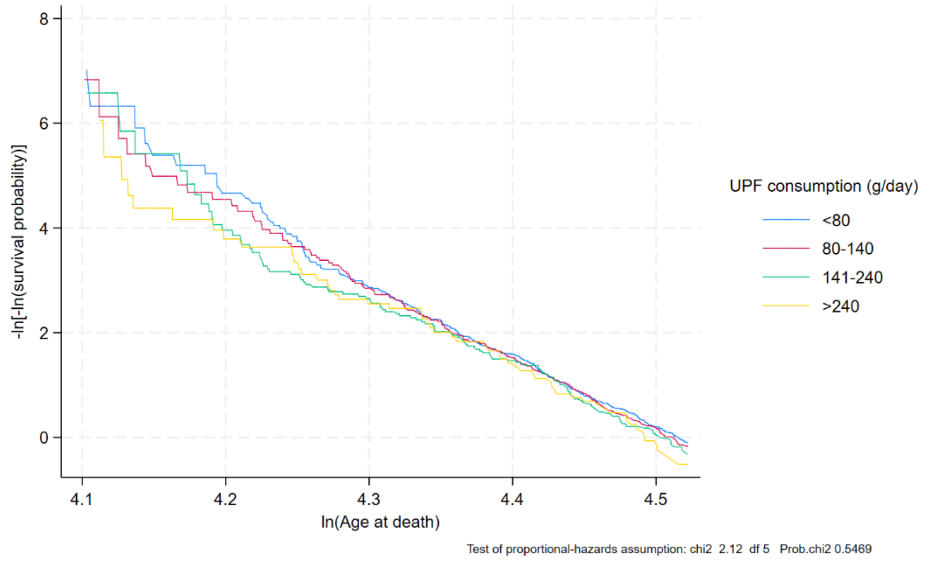

Figure S1. Proportional-hazards assumption by Ultra Processed Food consumption quartiles (g/day)

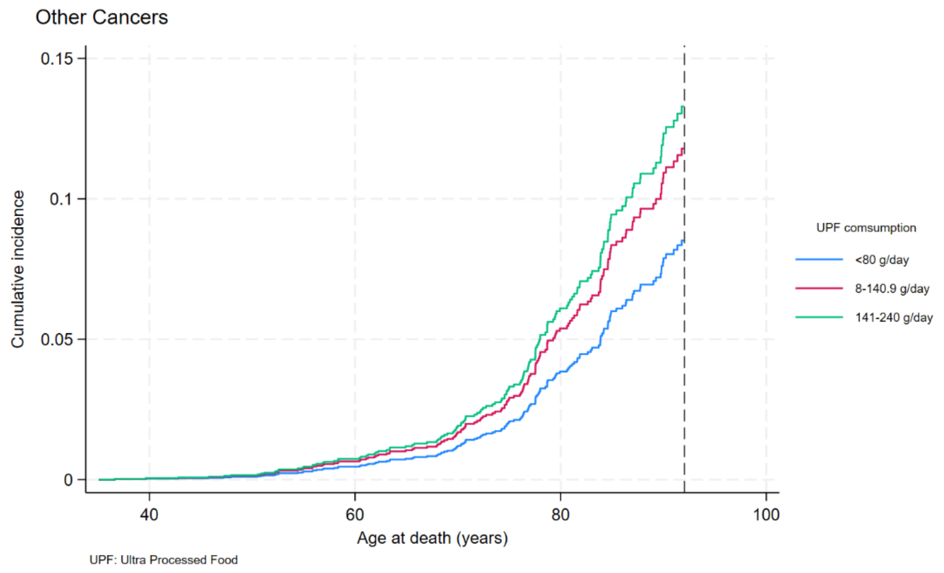

Figure S2. Cumulative Incidence for Other Cancer Death by Ultra Processed Food consumption quartiles (g/day)
